# Phytochemical profiles and biological activity of *Myrsine africana* L.: a comprehensive review

**DOI:** 10.3389/fphar.2025.1565656

**Published:** 2025-03-20

**Authors:** Getaneh Worku Moges, Gizachew Mulugeta Manahelohe, Melesse Ababay Assege, Banchamlak Sewachen Tasew, Desilal Kokebie Molla, Aderaw Anteneh Belew

**Affiliations:** ^1^ Department of Chemistry, College of Natural and Computational Sciences, University of Gondar, Gondar, Ethiopia; ^2^ Department of Biology, College of Natural and Computational Sciences, University of Gondar, Gondar, Ethiopia; ^3^ Department of Chemistry, College of Natural and Computational Sciences, Jigjiga University, Jigjiga, Ethiopia

**Keywords:** *Myrsine africana*, biological activity, antioxidant activity, antibacterial activity, secondary metabolites

## Abstract

*Myrsine africana* L. is a member of the Myrsinaceae family, which encompasses more than 1,000 species and 35 genera predominantly found in tropical and subtropical regions. This plant is abundant in Africa and Asia, and has been traditionally utilized for its aromatic properties in tea, spices, appetizers, carminatives, and flavoring agents. Despite its wide-ranging applications, a comprehensive review of its phytochemical potential and biological effects has not yet been conducted. This study aims to fill that gap by reviewing the phytochemical composition and biological activities of *M. africana*. Literature was gathered using databases such as Google Scholar, PubMed, Scopus, and Web of Science. The wide range of uses of *M. africana* can be attributed to its rich phytochemicals, including alkaloids, flavonoids, phenols, terpenoids, and saponins. Among its significant biological activities, *M. africana* is known for its anti-inflammatory and antioxidant properties. Furthermore, it shows potential in antispasmodic, antityrosinase, antibacterial, anti-aging, and anticancer applications. Additionally, it is used to treat conditions, such as malaria, helminthosis, wounds, tuberculosis, and gastrointestinal complications. Some of the isolated compounds from different parts of *M. africana* include methylvilangin **(11)**, methylanhydrovilangin **(12)**, 2-hydroxychrysophanol **(13)**, myrsinene **(25)**, myrsigenin **(26)**, myrsininone A **(27)**, myrsininone B **(28)**, and myrsinoside B **(30)**, as well as various other flavonoid compounds. This review aims to systematically explore the phytochemical profiles and associated biological activities of *M. africana*, highlighting key compounds and their pharmacological implications. By bringing together information, it emphasizes the potential of *M. africana* in drug discovery and future research.

## 1 Introduction

Medicinal plants have broad applications for preventing and treating a variety of illnesses. Natural products and their derivatives are essential to modern medications and lead compounds in developing new drugs (Abbhi et al., 2016). In many underdeveloped nations, the majority of people still depend on the use of medicinal plants for their healthcare needs. This percentage may be as high as 85% in sub-Saharan Africa and approximately 70%–80% among Africans. With their low cost, easy accessibility, and few side effects, medicinal plants may provide fresh hope for the development of alternative medications for a variety of disorders (Getachew et al., 2022).

*Myrsine africana* L. is a member of the Myrsinaceae family, a large family with more than 1,000 species and 35 genera, mostly found in tropical and subtropical regions. It is extensively available in Africa and Asia. The evergreen shrub *M. africana* grows slowly to a height of 2 m. African boxwood and Cape myrtle are popular names for *M. africana*. Traditionally, the plant is used for its fragrance in tea, spice, appetizer, carminative, and to flavoring agents. Its fruits are consumable and locally used as an anthelmintic and to treat conditions like diarrhea, rheumatism, toothache, lung conditions, tuberculosis, and hemorrhage (Ahmad et al., 2011c; Gul et al., 2021; Laraib et al., 2021).

Despite growing interest in the therapeutic potential of *M. africana*, research on its bioactive compounds and pharmacological effects remains fragmented. Secondary metabolites such as flavonoids, alkaloids, phenols, and tannins have demonstrated significant biological and pharmacological activities. *Myrsine africana* has shown a range of bioactive effects, including hemagglutination, anthelmintic, insecticidal, and anti-inflammatory properties, as well as the ability to lower blood glucose levels (Amare, 2021; Laraib et al., 2021). *Myrsine africana* exhibits relatively high antioxidant and antibacterial activity, with relatively high quantities of total flavonoids, phenols, and tannins in methanolic fruit extracts (Gul et al., 2021). The plant methanol extract of the shoot exhibits antioxidant and antityrosinase activities (Kishore et al., 2018), the methanolic and hydroalcoholic extracts also demonstrate anti-inflammatory and analgesic properties (Abbhi et al., 2016), while the fruit and leaf extracts have demonstrated antibacterial properties (Ahmad et al., 2016).

Moreover, the recently isolated compound myrsinene from aerial parts of *M. africana* possesses anti-inflammatory activity (Ahmad et al., 2011a), while Kishore et al. (2018) explored the isolation of flavonoid compounds with antityrosinase and antioxidant activity. Additionally, myrsigenin isolated from the methanol extract, demonstrated its antispasmodic properties (Azam et al., 2011). This review aims to provide scientific insights into the phytochemical profiles, biological activity, and isolated compounds of *M. africana*.

While many studies have explored aspects of *M. africana*, such as its chemical composition and bioactive compounds, no comprehensive review has yet synthesized these findings. This review aims to bridge that gap by providing a detailed overview of the plant’s phytochemical profiles, biological activities, and medicinal properties. Additionally, it highlights underexplored areas and suggests future research directions, particularly in the formulation of new therapeutic agents. By consolidating existing knowledge, this review underscores *M. africana*’s potential in drug discovery and its importance in medicinal research.

## 2 Materials and methods

A comprehensive literature search was conducted in science databases, like PubMed, Scopus, Google Scholar, and Web of Science, to identify studies focused on the phytochemical profiles, biological activities, and ethnopharmacology of *M. africana*. The search was performed using keywords such as “*M. africana*,” “phytochemical profiles,” “bioactive compounds,” “antibacterial activity,” “anti-inflammatory effects,” and “antioxidant activity.” Boolean operators (AND, OR) were used to refine the search, and the literature was limited to studies published until the end of 2024. Inclusion criteria were limited to peer-reviewed articles in English that focused on identifying phytochemicals and biological activities of *M. african*a while excluding non-peer-reviewed sources and studies not directly related to the plant’s medicinal properties.

Data from the selected studies were extracted on the phytochemical compounds identified in *M. africana* and their associated biological activities, including antioxidant, antibacterial, antifungal, anti-inflammatory, and cytotoxic activity. The findings were synthesized to provide a comprehensive overview of the plant’s medicinal potential. The chemical structure of bioactive compounds was drawn using ChemDraw ultra 8.0 software. This review follows a systematic review approach, aiming to summarize the current understanding of *M. africana* phytochemical composition and pharmacological activities while highlighting areas for future research.

## 3 Ethnopharmacology of *Myrsine africana*

*Myrsine africana* has a storied history of traditional use in diverse medicinal applications. An ethnopharmacological survey exhaustively explored by various researchers in the literature is comprehensively presented in Table 1.

**TABLE 1:** Ethnopharmacology of the different parts of *Myrsine africana*.

Plant part	Origin	Condition	Pharmacological effects/Medicinal uses	References
Fruit	Pakistan	Methanol extract	Antioxidant, Antimycobacterium tuberculosis, antibacterial activities and cytotoxicity	Gul et al. (2021)
		Methanol and chloroform extracts	Cytotoxic, antibacterial, antifungal, antitumor, and antioxidant properties	Laraib et al. (2021)
	Ethiopia	NA	Utilized as food and medication to treat childhood ascaris	Ashagre et al. (2016)
			Used to treat Intestinal parasites	Tahir et al. (2023)
	India	Methanol and Hydro-Alcoholic Extract	Analgesic, and Anti-inflammatory Activity	Abbhi et al. (2016)
	Kenya	Ethanol 9:1 water extract	Antibacterial activity	Obey et al. (2016)
		NA	No significant anthelmintic activity was observed against *Haemonchus contortus* in sheep treated with a dose of 50 g per animal	Githiori et al. (2002)
Leaf	South Africa	Ethanol extract and isolated compound myrsinoside B	*In vivo* and *in vitro* Elastase Inhibition and Anti-wrinkle Activity	Lall et al. (2017)
		Crude ethanol extract and its isolated compound myrsinoside B	*In Vitro* Anti-inflammatory, Antioxidant, and Skin Permeation	Fibrich et al. (2019)
		Ethanol extract	Anthelmintic, anti-acne, and tyrosinase inhibiting activity	Stapelberg et al. (2019)
	Ethiopia	Methanol	Antidyslipidemic and antihyperglycemic effects in alloxan-induced diabetic mice	Amare (2021)
		Crude aqueous and methanol extracts	Anti- tuberculosis	Getachew et al. (2022)
	Pakistan	Aqueous, methanol, ethanol, chloroform and n-hexane extracts using Soxhlet	*In vitro* antioxidant and antiproliferative, and *in vivo* hepatoprotective activity	Gul et al. (2017)
		Aqueous	Antioxidant, Antibacterial and Phytotoxic Activities of green-synthesized silver nanoparticles	Sarwer et al. (2022)
		Methanol and chloroform extracts	Cytotoxic, antibacterial, antifungal, antitumor, and antioxidant properties	Laraib et al. (2021)
	Kenya	NA	No significant anthelmintic activity was observed against *Haemonchus contortus* in sheep treated with a dose of 125 g per animal	Githiori et al. (2002)
Seed	Kenya	NA	Used to treat Malaria, Helminthosis, Wounds, Tuberculosis, Gastrointestinal complications	Nanyingi et al. (2008)
		Aqueous extract	Acute toxicity studies in male Wistar rats on some hematological and biochemical parameters	Kabubii et al. (2015)
		Aqueous, methanol, and n-hexane extracts	Anti-malaria	Shopoko et al. (2020)
		NA	Effectiveness of anthelmintics against mixed gastrointestinal nematodes in sheep	Muthee (2018)
Root	China	Methanol	Cytotoxic, antioxidant, apoptotic, and wound-healing characteristics of green-synthesized silver nanoparticles	Ahn et al. (2019)
Shoot	South Africa	Crude Methanol Extract, its fraction and isolated Flavonoids and Flavonoid Glycosides compounds	Antioxidant and Antityrosinase Activity	Kishore et al. (2018)
Stem	China	Isolated compound myrsininone A, and myrsininone B	Antibacterial	Kang et al. (2007)
Aerial parts	Pakistan	Crude methanol extract	Anti-spasmodic action	Azam et al. (2011)
		Crude methanol extract and its fractions	Phytotoxic, Hemagglutination, and Antibacterial activities	Ahmad et al. (2011c)
		Methanol extract and their fractions	Antibacterial activity	Ahmad et al. (2016)
		Crude methanol extracts, and its fractions	Anti-inflammatory activity	Ahmad et al. (2011a)
		Crude methanol extract and its fractions	*In vitro* antifungal, insecticidal, Brine shrimp cytotoxicity and antioxidant activity	Ahmad et al. (2011b)
Raw ripe fruits	Ethiopia	NA	Food, toothbrush	Hankiso et al. (2023)

NA, not available.

## 4 Phytochemical profiles and isolated compounds from *Myrsine africana*

### 4.1 Identified phytochemicals

*Myrsine africana* is known to contain a diverse array of phytochemicals, including alkaloids, phenolic compounds (including flavonoids, phenolic acids, and tannins), terpenoids, and saponins (Abbhi et al., 2011; Amare, 2021; Gul et al., 2017; Laraib et al., 2021; Shopoko et al., 2020). These compounds contribute to the medicinal properties and biological activities of this plant (Mehmood et al., 2024). Researchers have investigated the phytochemical profiles of *M. africana* extracts using various solvents and methods to understand the effect of solvents on the yield and composition of bioactive compounds.

Phytochemicals are naturally occurring chemical compounds synthesized by plants that offer various health benefits to consumers. Many of these compounds protect the human body from oxidative stress, inflammation, and various diseases when fruits and vegetables are consumed regularly (Gul et al., 2021).

A high-quality medicinal plant extract should contain a significant amount of phytochemicals. The extracts from *M. africana* were found to be particularly rich in a wide range of phytochemicals, as shown in Table 2. The selection of extraction solvent had an impact on the levels of flavonoids, alkaloids, and terpenoids present in the extracts (Amare, 2021; Gul et al., 2017; Gul et al., 2021; Laraib et al., 2021). Analysis revealed that the seeds of *M. africana* were found to contain high concentrations of phenols, tannins, steroids, and saponins in their aqueous, methanol, and n-hexane extracts. Alkaloids were identified in the aqueous and methanol extracts, flavonoids were exclusively present in the aqueous extract, and terpenoids were detected only in the methanol extracts (Shopoko et al., 2020). In another study by Gul et al. (2017), it was supported that the methanol extracts of the leaves of *M. africana* contained greater amounts of phenols, flavonoids, alkaloids, saponins, and tannins than the ethanol, chloroform, and aqueous extracts. Notably, the presence of these bioactive compounds from *M. africana* has demonstrated particular efficacy against diseases, highlighting the plant’s medicinal value. Alkaloids from *M. africana* have demonstrated particular efficacy against malaria (Shopoko et al., 2020).

**TABLE 2:** Identified phytochemicals in different parts of *Myrsine africana* plant and solvent system.

Plant parts	Origin	Solvents	Extraction method	Identification method	Identified phytochemicals	References
Seed	Kenya	Aqueous	Maceration	QT	Phenols, tannins, Alkaloids, steroids, saponins, and flavonoids	Shopoko et al. (2020)
		Methanol	Maceration	QT	Phenols, tannins, Alkaloids, steroids, saponins, and terpenoids	
		n-hexane	Maceration	QT	Phenols, tannins, steroids, and saponins	
Fruits	India	Methanol	Soxhlet	QT	Reducing sugars, tannins, flavonoids, amino acids, and saponins	Abbhi et al. (2011)
		Aqueous	Decoction	QT		
Fruit and leaf	Pakistan	Methanol and chloroform	Maceration	QT	Flavonoids, carbohydrates, terpenoids, steroids, tannins, and saponins	Laraib et al. (2021)
Leaf	Ethiopia	Methanol	Maceration	QT	Flavonoids, terpenoids, glycosides, tannins, steroids, saponins, phenols, and alkaloids	Amare (2021)
	Pakistan	Methanol	Soxhlet	HPLC	Rutin, quercetin, and p-coumaric acid	Gul et al. (2017)
				GC-MS	Ethanol (25.95%), octadecenoic acid (24.36%), ascorbic acid (24.26%), heptacosanol (6.07%), stearic acid (5.59%), heptacosylheptafluorobutyrate (3.60%), hexacontane (3.28%), palmitosyl chloride (3.02%), phosphaheptacos (2.14%) and isobutyl alcohol (1.73%)	

GC-MS, gas chromatography‒mass spectrometry; HPLC, high-performance liquid chromatography analysis; and QT, qualitative test.

The study conducted by Gul et al. (2017) revealed significant findings regarding the phytochemical profiles of methanol extracts of *M. africana* leaves through high-performance liquid chromatography (HPLC) analysis, which contains the most prominent compounds: rutin and quercetin: a flavonoid compound known for its antimicrobial, antioxidant, anti-inflammatory, antidiabetic, and anticancer activities (Gullón et al., 2017; Vollmannová et al., 2024), as well as p-coumaric acid, is a phenolic compound with a range of biological activities such as antioxidant, anti-inflammatory, and antimicrobial activities (Memar et al., 2020). These findings emphasize the bioactive potential of *M. africana* methanol extracts and their relevance for therapeutic applications.

The choice of extraction solvent plays a significant role in the efficiency of extracting various phytochemicals in *M. africana*. Methanol is notably more effective at extracting diverse phytochemicals, whereas ethanol yields moderate amounts for most compounds, though it is less effective than methanol. Aqueous solutions contain flavonoids and saponins, but these are present in significantly lower concentrations than organic solvents. Chloroform does yield detectable amounts of these compounds, but its extraction yields are lower than methanol and ethanol. In solvent extraction, choosing the appropriate solvent is crucial, considering factors such as selectivity, solubility, and safety. Solvents that share a polarity similar to the solute typically yield better results (Lee et al., 2024). The leaf extract demonstrated a greater bioactive constituent than the seeds and fruits, underscoring the importance of plant part selection. However, the variation in yield depending on the part of the plant and extraction methods suggests that tailored extraction protocols and solvent compositions for *M. africana* are essential for optimizing specific compounds with broad medicinal relevance.

The amount and types of compounds extracted from plants, as well as the biological activity of the extracts, are significantly influenced by the extraction method used. Previous studies on *M. africana* have utilized both Soxhlet and maceration techniques for extracting bioactive compounds. Soxhlet extraction is known for its efficiency, as it uses continuous solvent circulation to extract compounds from plant material at elevated temperatures, making it ideal for extracting alkaloids, flavonoids, and tannins (Abbhi et al., 2011; Gul et al., 2017). However, while Soxhlet results in higher yields, it also poses the risk of degrading heat-sensitive compounds such as polyphenols, flavonoids, and alkaloids during the thermal extraction process, which can lead to a reduction in biological activity. On the other hand, maceration, a simpler technique, involves soaking the plant material in a solvent at room temperature over an extended period. Though gentler on heat-sensitive compounds and better at preserving their biological activity, maceration is less efficient and requires more time (Bitwell et al., 2023; Mungwari et al., 2024). Studies by Gligor et al. (2023b) on green coffee beans and *Xanthium spinosum L.* demonstrate that Soxhlet extraction yields the highest levels of bioactive compounds, particularly polyphenolics (Gligor et al., 2023a).

In recent years, modern extraction methods such as ultrasound-assisted extraction (UAE), pressurized liquid extraction (PLE), supercritical fluid extraction (SFE), and microwave-assisted extraction (MAE) are increasingly favored due to their higher efficiency, lower energy consumption, reduced solvent use, and environmentally friendly nature (Mungwari et al., 2024). These methods enable faster extractions while preserving the structure and bioactivity of bioactive compounds. For instance, UAE uses ultrasonic waves to improve solvent penetration and increase the efficiency of compound release, resulting in higher yields compared to traditional methods (Shen et al., 2023). MAE utilizes microwave radiation to heat the solvent, which improves the extraction process while minimizing thermal degradation and preserving the biological activity of the compounds. SFE uses high pressure and a supercritical fluid (usually CO_2_) to extract bioactive compounds, maintaining the integrity of sensitive compounds and preserving their biological efficacy (Mungwari et al., 2024). Overall, while traditional methods like Soxhlet and maceration can be effective, modern extraction techniques such as MAE and UAE are recommended for their efficiency, minimal solvent use, and superior preservation of biological activity. To improve the efficiency of bioactive compound extraction from *M. africana*, further research is recommended on modern extraction methods to optimize yield and preserve bioactivity.

**FIGURE 1:**
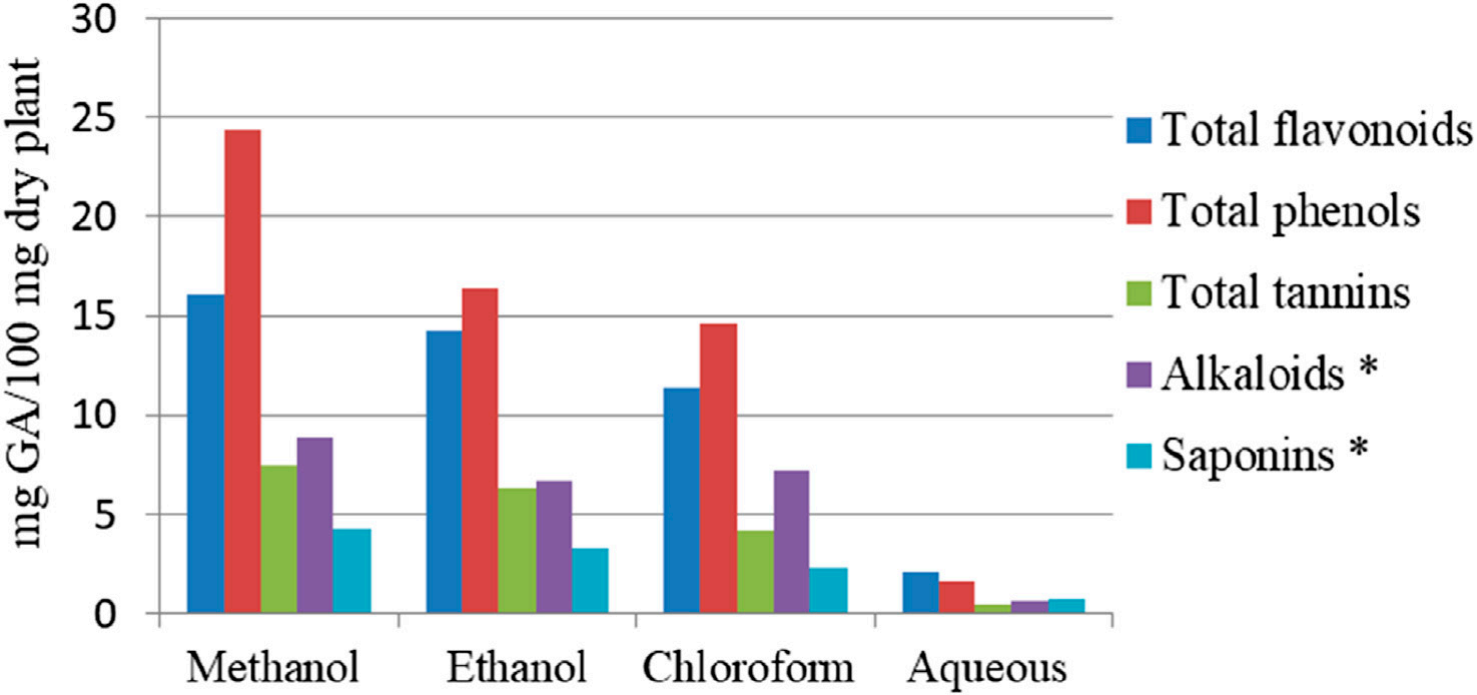
Total flavonoid, phenol, tannin, alkaloid, and saponin contents of the leaf extracts of *Myrsine africana* (* represents in terms of mg/100 mg dry plant) Gul et al. (2017).

**FIGURE 2:**
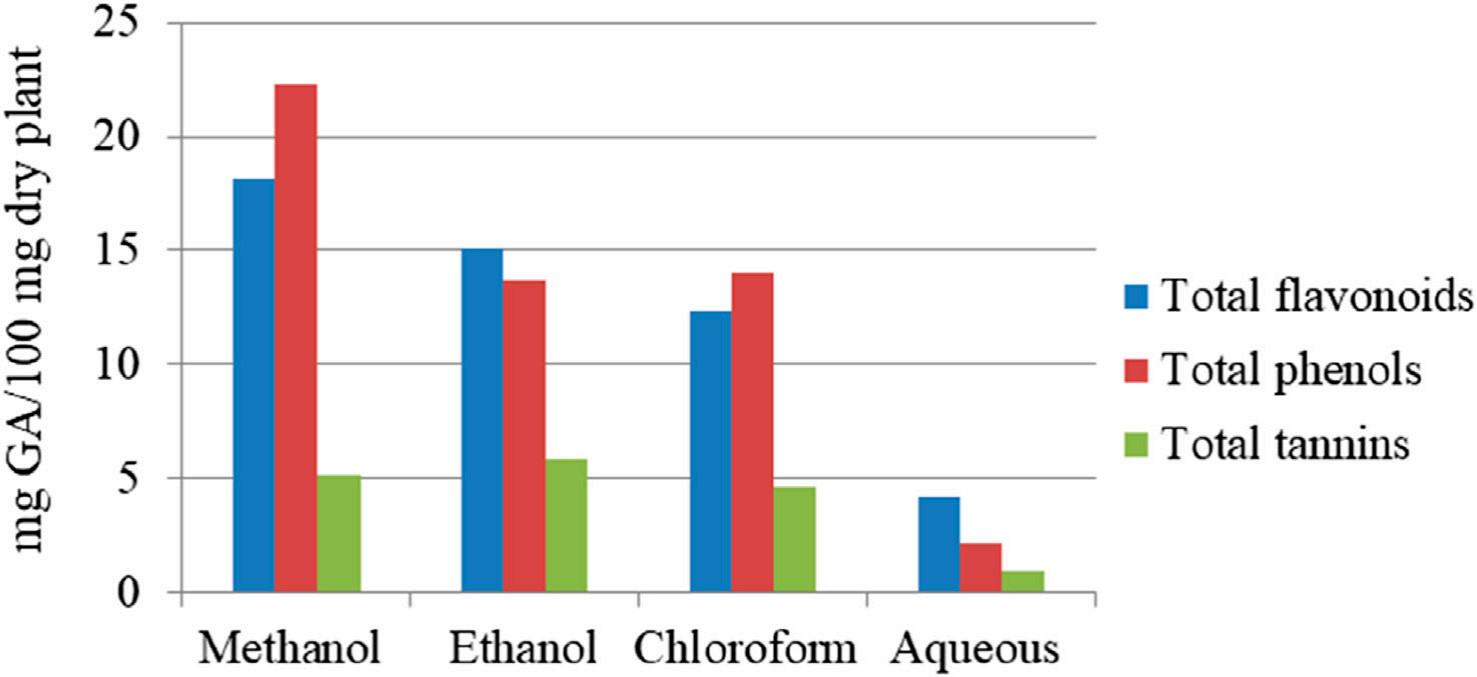
Total flavonoid, phenol, and tannin contents of the fruit extracts of *Myrsine africana* Gul et al. (2021).

The quantitative analysis of *M. africana* reveals significant variability in phytochemical content depending on the plant part and solvent used for extraction, with methanol consistently emerging as the most effective solvent and aqueous being the least, as shown in Figures 1, 2. Phenolic content ranged from 0.089 mg/g to 40.34 ± 2.51 mg gallic acid equivalent (GAE)/g of dried plant (Amare, 2021; Gul et al., 2017; 2021; Laraib et al., 2021), with methanol leaf extracts showing the highest levels of phenolics (40.34 ± 2.51 mg GAE/g) and flavonoids (36.28 ± 4.13 mg quercetin equivalent (QE)/g) (Amare, 2021). Similarly, the methanol fruit extract demonstrated high concentrations of saponins (140 mg/g) (Laraib et al., 2021), alkaloids (8.89 ± 0.25 mg/100 g), and tannins (7.53 ± 0.38 mg GA/100 g) (Gul et al., 2017). Abbhi et al. (2011) also found that the fruit methanolic extract contains 17.5% saponins and 4% tannins. These findings highlight methanol’s efficacy in extracting a wide range of bioactive compounds, making it the optimal solvent for maximizing the therapeutic potential of *M. africana* in medicinal applications.

### 4.2 Compounds isolated from *Myrsine africana*

Secondary metabolites from plants, including alkaloids, flavonoids, and phenolic chemicals, can lower blood glucose levels (Amare, 2021). Numerous flavonoids, phenolic compounds, and terpenoids have been identified and isolated from *M. africana*. These compounds have significant applications in biological activities, such as anti-inflammatory, antioxidant, antispasmodic, antityrosinase, and antibacterial activities, as well as antiaging, anticancer, and preventive effects against neurodegenerative disease, cardiovascular disease, diabetes mellitus, and obesity (Azam et al., 2011; Fibrich et al., 2019; Kishore et al., 2018; Laraib et al., 2021).

**FIGURE 3:**
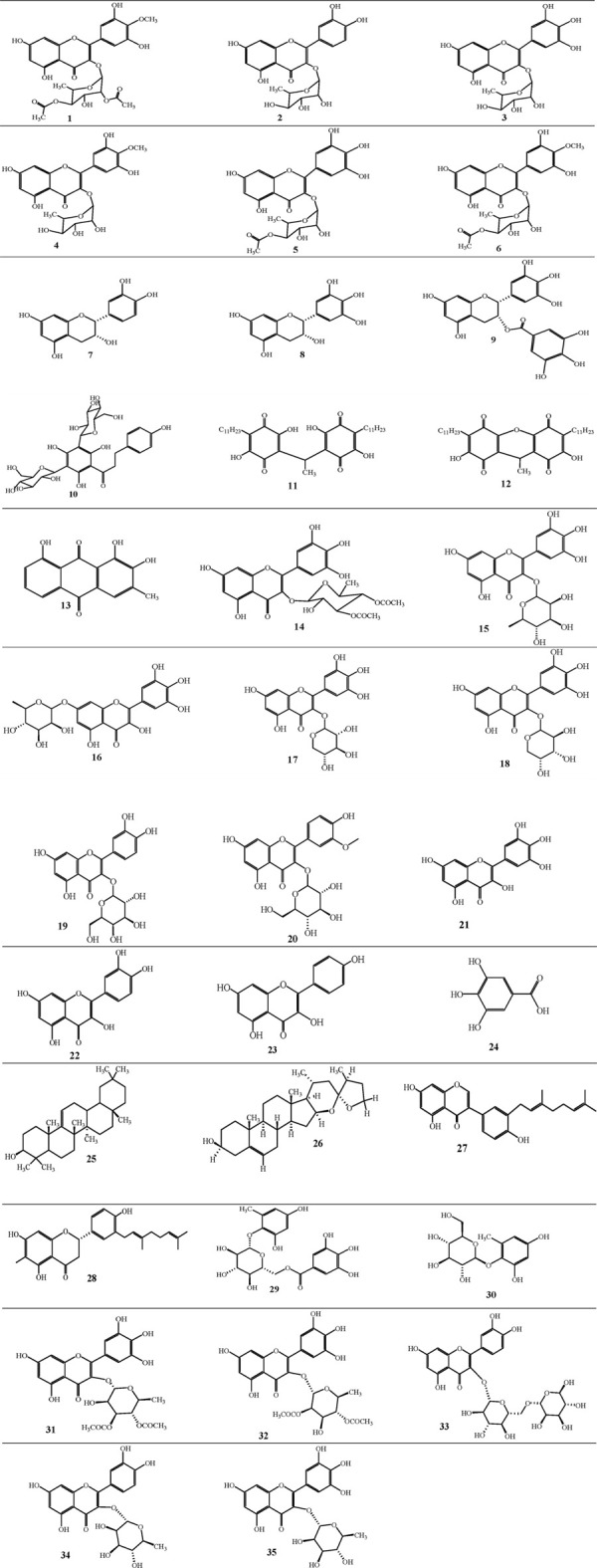
Structures of the isolated compounds from different parts of *Myrsine africana*.

The isolated compounds, methods of the chromatographic techniques used for isolation, methods of characterization of compounds and solvents used to extraction from different parts of *M. africana* are summarized as shown in Table 3. These compounds further contribute to the biological activity of *M. africana*, reinforcing its potential in medicinal applications. The structures of the isolated compounds are shown in Figure 3.

**TABLE 3:** Isolated compounds, methods used for isolation and characterization, and solvents used for extraction.

Isolated compounds	Plant parts used and extraction solvent	Chromatographic techniques used for isolation of compounds	Characterization methods	References
Mearnsetin 3-(2″,4″-diacetylrhamnoside) **(1)**, quercitrin **(2)**, myricitrin **(3)**, mearnsitrin **(4)**, myricetin-3-O-(4″-O-acetyl)-a-L-rhamno- pyranoside **(5)**, mearnsetin-3-O-(4″-O-acetyl)-a-L-rhamno- pyranoside **(6)**, (−)-epicatechin **(7)**, (−)-epigallocatechin **(8)**, (−)-epigallocatechin-3-O-gallate **(9)**, and 3′,5′-di-C-β- glucopyranosyl phloretin **(10)**	Ethanol extract of the stem	Column chromatography	HR- ESI-MS, ^1^H and^13^C NMR, IR, HMBC, and DEPT-NMR	Zou et al. (2009)
Methylvilangin **(11)** and methylanhydrovilangin **(12)**	Ethyl acetate extract of the fruit	Column and thin layer chromatography	IR, EIMS, ^1^H and^13^C- NMR, NOESY, HMQC, and HMBC	Manguro et al. (2003)
2-hydroxychrysophanol **(13)**	Ethanol extract of the root	Column and thin layer chromatography	IR, EIMS, ^1^H-NMR, and UV	Li and McLaughlin (1989)
Myricetin 3-(3″,4″- diacetyl-a-L-rhamnoside) **(14)**, myricetin 3-rhamnoside **(15)**, myricetin 7-rham- noside **(16)**, myricetin 3-xyloside **(17)**, myricetin 3-arabinoside **(18)**, quercetin 3-galactoside **(19)**, isorhamnetin 3- glucoside **(20)**, myricetin **(21)**, quercetin **(22)**, kaempferol **(23)** and gallic acid **(24)**	Methanol extract of the leaf	Column, flash, and thin layer chromatography	IR, EIMS, ^1^H and^13^C NMR, and UV	Arot et al. (1996)
Myrsinene **(25)**	Methanol extract of the areal part	Column and thin layer chromatography	^1^H and^13^C-NMR, HREI-MS, HSQC, HMBC, COSY and NOESY	Ahmad et al. (2011a)
Myrsigenin **(26)**	Methanol extract of the areal part	Column and thin layer chromatography	HREI-MS, UV, ^1^H and^13^C-NMR,COSY, HSQC, HMBC and NOESY	Azam et al. (2011)
Myrsininone A **(27)** (an isoflavone) and myrsininone B (**28)** (a flavanone), exhibited remarkable antibacterial activities	Ethanol extract of the stem	Column chromatography	IR, EIMS, ^1^H and^13^C-NMR, COSY, and HMBC	Kang et al. (2007)
Mearnsitrin **(4)**, myricetin 3-O-(4″-O-acetyl)-α-L-rhamnopyranoside **(5)**, mearnsetin 3-O-(4″-O-acetyl)-α-L-rhamnopyranoside **(6)**, myricetin **(21)**, quercetin **(22)**, myrsinoside A **(29)**, myrsinoside B **(30)**, quercetin-3-(3″,4″-di-O-acetyl)-α-L-rhamnoside **(31)**, myricetin-3-O-(2″,4″-di-O-acetyl)-α-L-rhamnopyranoside **(32)**, rutin **(33)**, quercetin 3-O-α-L-rhamnopyranoside **(34)**, and myricetin 3-O-α-L-rhamnopyranoside **(35)**	Methanol extract of the shoot	Column and thin layer chromatography	UV, IR, HR-MS, ^1^H and^13^C-NMR, COSY, HSQC, and HMBC	Kishore et al. (2018)

COSY, correlation spectroscopy; DEPT, distortionless enhancement by polarization transfer; HMBC, heteronuclear multiple bond correlation; HMQC, heteronuclear multiple quantum coherence; HREI-MS, high-resolution electron ionization mass spectrometry; HSQC, heteronuclear single quantum correlation spectroscopy; IR, infrared spectroscopy; NMR, nuclear magnetic resonance spectroscopy; NOESY, nuclear overhauser effect spectroscopy; UV, ultraviolet.

## 5 Biological activity

### 5.1 Antioxidant activity

**FIGURE 4:**
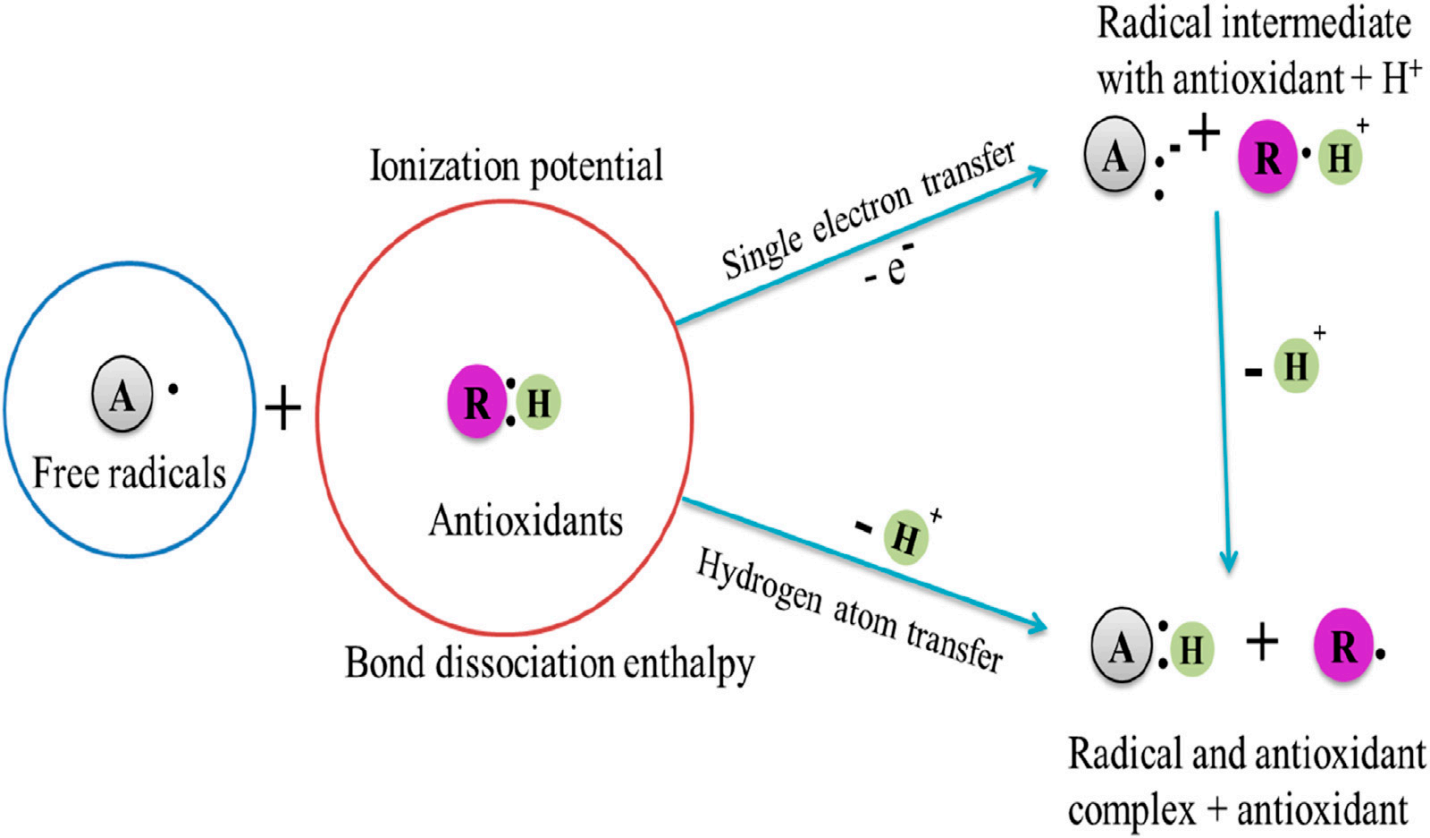
The pathway of interactions between antioxidant and free radical Siddeeg et al. (2021).

Antioxidants are substances that prevent autoxidation by blocking or halting the formation and proliferation of free radicals. Their mode of action includes hydrogen atom transfer, single electron transfer, and chelation of transition metals (Moges et al., 2024). Understanding how antioxidants interact with free radicals is essential for protecting our health, as this mechanism plays a crucial role in protecting our bodies from oxidative stress and preventing cellular damage (Figure 4).

**FIGURE 5:**
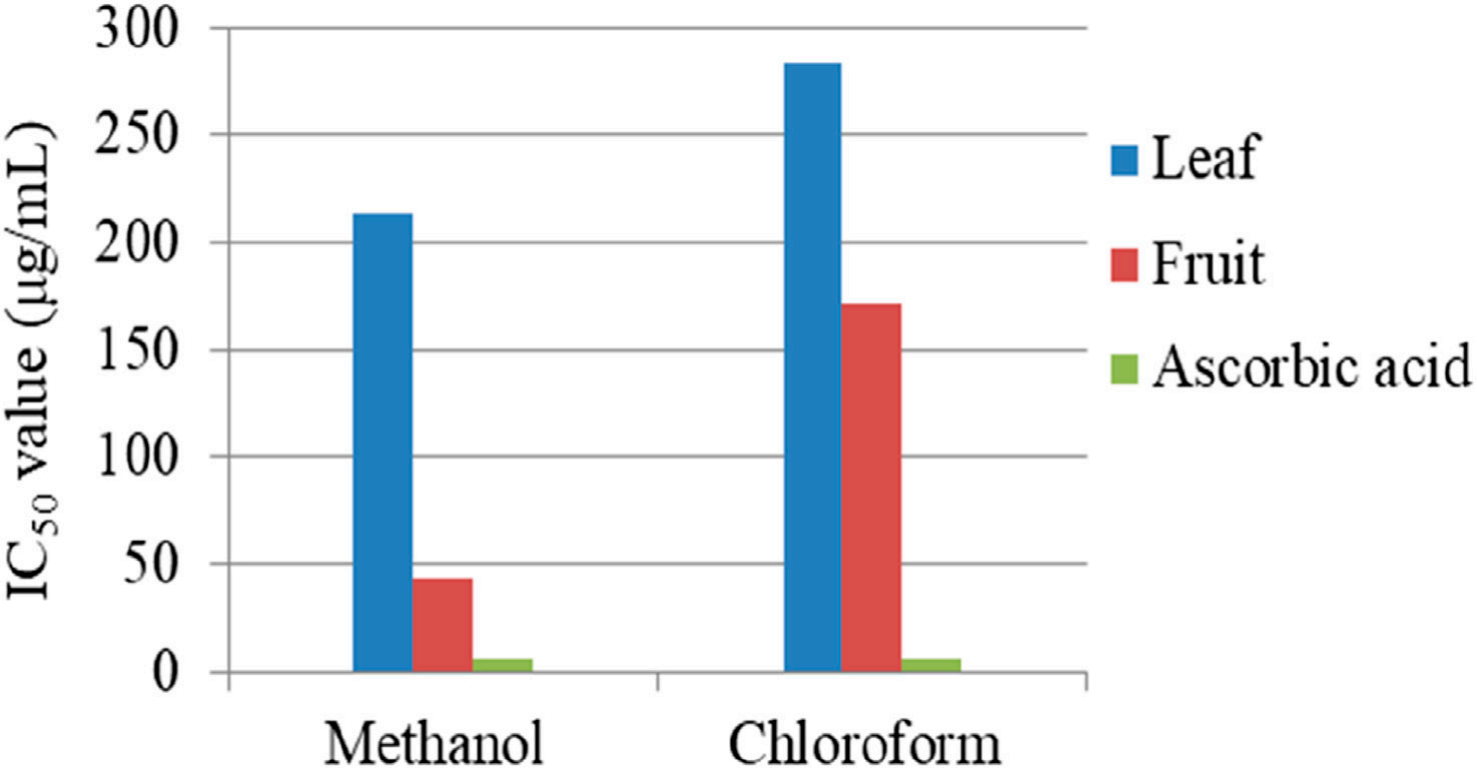
Antioxidant activity of *Myrsine africana* fruit and leaf extracts Laraib et al. (2021).

**FIGURE 6:**
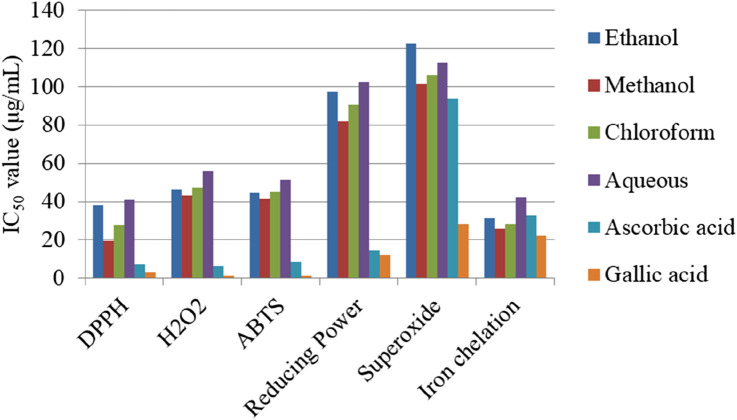
Antioxidant activity of *Myrsine africana* leaf extract Gul et al. (2017).

**FIGURE 7:**
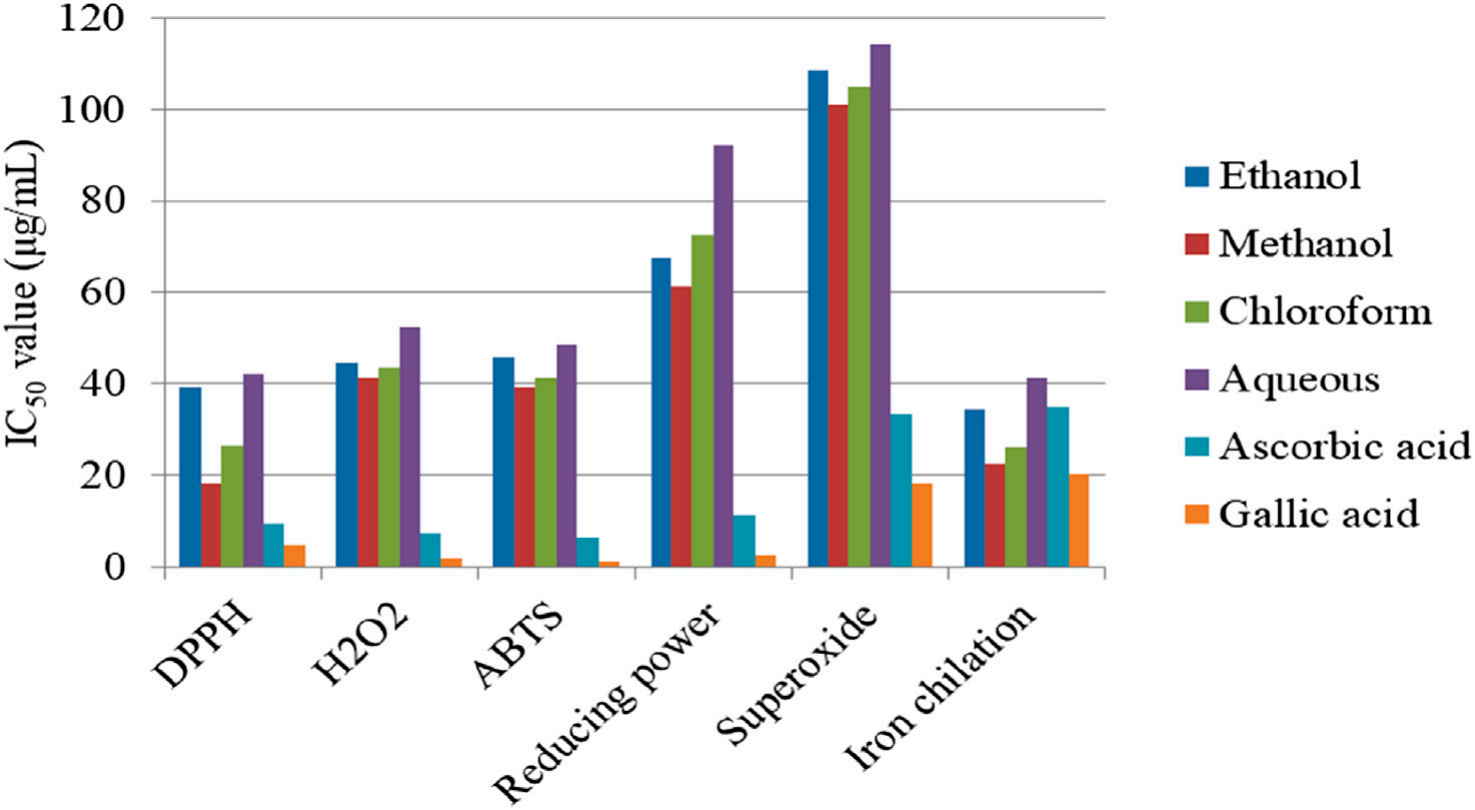
Antioxidant activity of *Myrsine africana* fruit extract Gul et al. (2021).

*Myrsine africana* is a rich source of alkaloids, terpenoids, flavonoids, steroids, and phenols, known for their potential antioxidant properties. Laraib et al. (2021) evaluated the antioxidant activity of *M. africana* fruits and leaves using methanol and chloroform extracts through a DPPH assay. The fruits showed more effective antioxidant activity than the leaves, with IC50 values of 43.84 μg/mL (methanol) and 171 μg/mL (chloroform) for fruits compared to 214 μg/mL (methanol) and 284 μg/mL (chloroform) for leaves, as shown in Figure 5. All samples had lower antioxidant concentrations than ascorbic acid (5.8 μg/mL). Methanol extracts were more effective at scavenging free radicals than chloroform extracts. In the phosphomolybdate assay, higher absorbance values indicate higher antioxidant activity. Methanolic fruit extracts exhibited the highest absorbance, ranging from 0.677 to 0.159 nm, while chloroform fruit extracts showed absorbance values from 0.502 to 0.176 nm, suggesting both extracts have significant antioxidant potential. In contrast, the methanol leaf extract ranged from 0.402 to 0.014 nm, and the chloroform leaf extract ranged from 0.414 to 0.013 nm, indicating lower antioxidant activity in leaf extracts. In contrast, the standard ascorbic acid exhibited much higher absorbance than the samples at all concentrations, ranging from 2.119 to 0.240 nm at 250 μg/mL and 7.81 μg/mL, respectively, confirming its superior antioxidant capacity. These findings highlight that fruit extracts, particularly the methanol extract, exhibit higher antioxidant activity than leaf extracts, with notable differences observed between methanol and chloroform extracts (Laraib et al., 2021). Similarly, Gul et al. (2017) and Gul et al. (2021) confirmed that methanol extracts consistently demonstrated higher antioxidant activity than chloroform, ethanol, and aqueous extracts, with aqueous being the least in all assay methods but lower activity than both ascorbic acid and gallic acid, as illustrated in Figures 6, 7. The antioxidant activity of methanol extract is higher as comparable with other solvent extracts due to methanol extracts higher amount of phenolic compounds, aqueous extract showed least activity which was linked to the low solubility of the bioactive constituents in water. Phenolic compounds principally phenols and flavonoids, which is a positive correlation with antioxidant activity (Abubakar et al., 2022). In a study by Amare (2021), the methanol extracts of the leaf of *M. africana* demonstrated strong antioxidant activity (IC_50_ = 75.32 μg/mL) using the DPPH assay. However, this activity is considerably weaker than ascorbic acid (IC_50_ = 13.21 μg/mL). The findings revealed that methanol was the most effective solvent for extracting compounds from *M. africana,* with the ability to scavenge free radicals and reactive oxygen species (ROS). A lower IC_50_ value indicates more effective antioxidant activity (Moges et al., 2024).

Gul et al. (2017) evaluated the antioxidant activity of the leaf extract of *M. africana* using six different methods, such as DPPH, ABTS, hydrogen peroxide (H_2_O_2_), reducing power, superoxide, and iron chelating, as illustrated in Figures 6, 7. Based on these findings, the DPPH method is the best method for the antioxidant activity of the *M. africana* plant, with lower IC_50_ values than other methods. Similarly, Gul et al. (2021) examined the fruit extracts and reported the same trend. The IC_50_ value of all extracts in DPPH, ABTS, and iron chelating assays is lower than 50 μg/mL, indicates that very strong antioxidant activity. The antioxidant activity levels were categorized according to IC_50_ values as follows: very strong (IC_50_ < 50 μg/mL), strong (IC_50_ between 50 and 100 μg/mL), medium (IC_50_ between 100 and 150 μg/mL), weak (IC_50_ between 150 and 200 μg/mL), and very weak (IC_50_ > 200 μg/mL) (Sengera et al., 2023).

**FIGURE 8:**
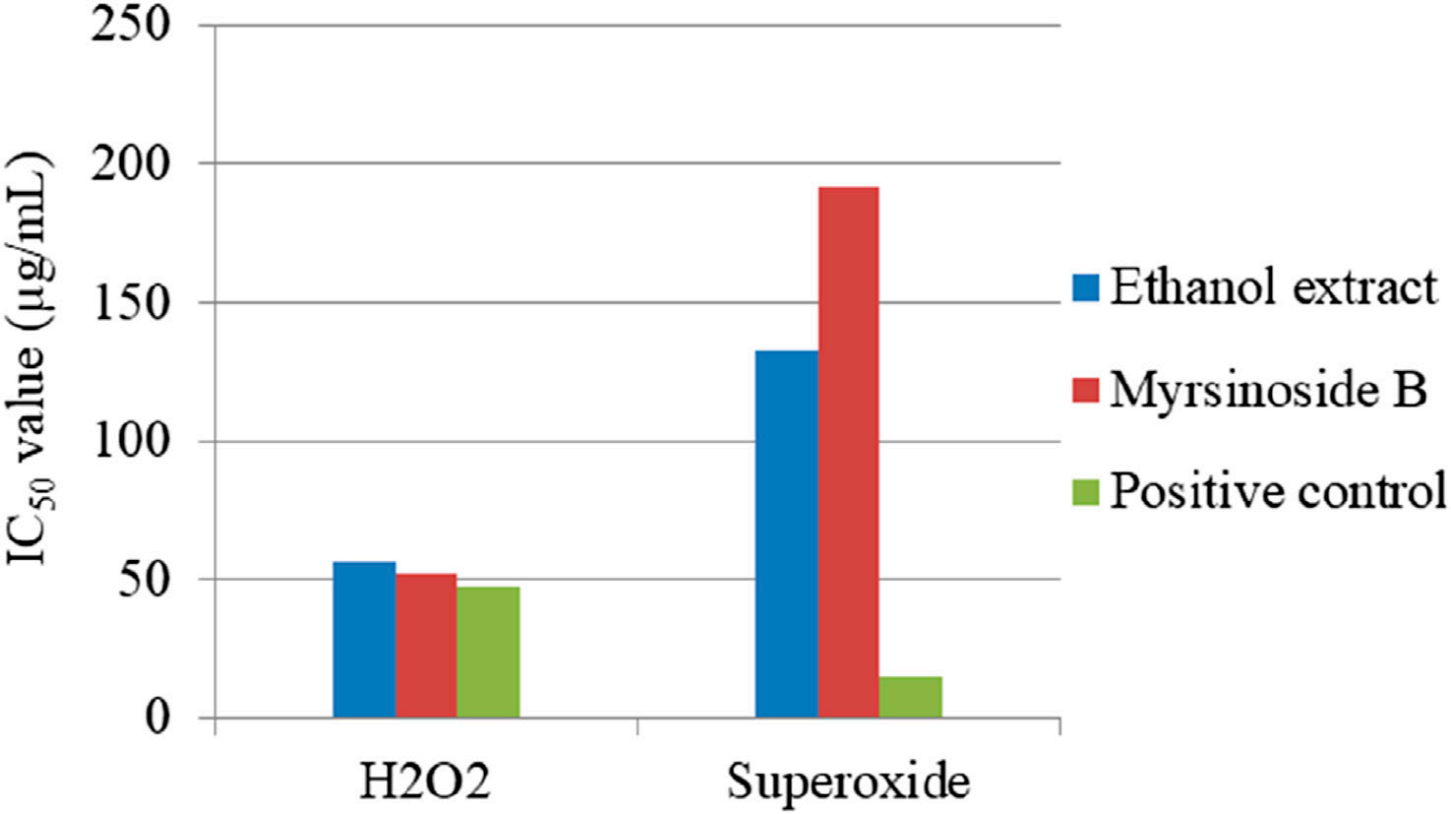
H_2_O_2_ and superoxide radical scavenging activity of the ethanol extract of *Myrsine africana* and its isolated compound myrsinoside B Fibrich et al. (2019).

**FIGURE 9:**
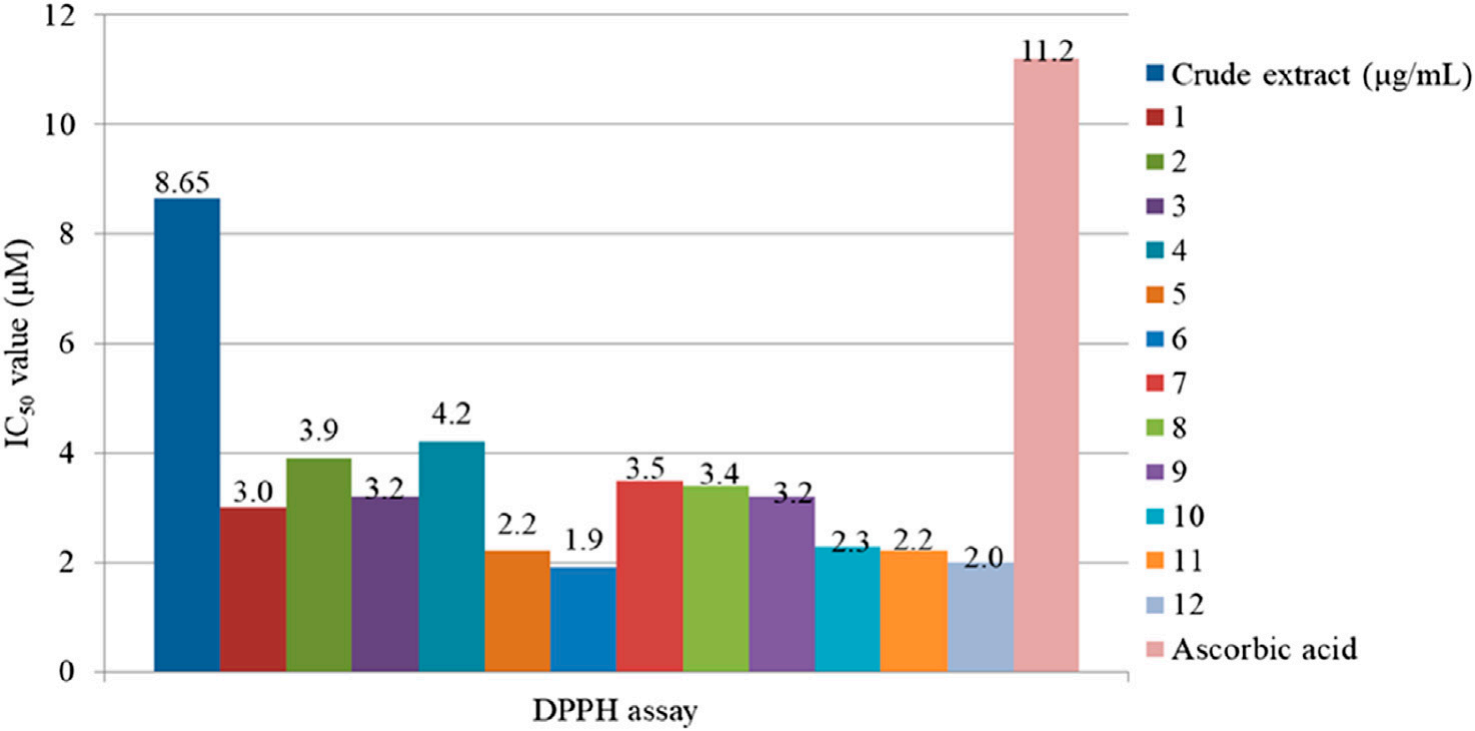
Antioxidant activity of the methanol extract of the shoot of *Myrsine africana* and the isolated compounds Kishore et al. (2018). Myrsinoside A (1), myrsinoside B (2), quercetin (3), myricetin (4), mearnsetin 3-O-(4′′-O-acetyl)-a-L- rhamnopyranoside (5), mearnsitrin (6), myricetin 3-O-(4′′-O-acetyl)-a-L-rhamnopyranoside (7), quercetin 3- (3′′,4′′-di-O-acetyl-a-L-rhamnoside) (8), myricetin 3-0-(2′′,4′′-di-O- acetyl)-a-L-rhamnopyranoside (9), rutin (10), quercetin 3-O-a-L- rhamnopyranoside (11), and myricetin 3-O-α-L-rhamnopyranoside (12).

The antioxidant activity of *M. africana* ethanol extracts and their isolated compound, myrsinoside B, has been extensively evaluated using superoxide and H_2_O_2_ assays (Fibrich et al., 2019). In the H_2_O_2_ assay, the crude ethanol extract exhibited an IC_50_ value of 56.08 ± 2.88 μg/mL, closely matched by the isolated compound myrsinoside B (52.19 ± 4.16 μg/mL), suggesting similar efficacy between the two (Figure 8). The ethanol extract and myrsinoside B show very strong antioxidant capacity, but their value is slightly higher than L-ascorbic acid (IC_50_ = 47.62 ± 3.34 μg/mL). On the other hand, the superoxide radical scavenging activity of the ethanol extract of *M. africana* (IC_50_ = 132.74 ± 1.64 μg/mL) was stronger than that of the isolated compound Myrsinoside B (IC_50_ = 192.14 ± 3.34 μg/mL), but both were significantly weaker than the positive control quercetin (15.29 ± 3.68 μg/mL) (Fibrich et al., 2019). These results highlight moderate antioxidant activity with limitations in potency compared to established standards. In another study, Kishore et al. (2018) also reported the antioxidant activity of methanol extract and isolated compounds from the shoot of *M. africana*. Based on these findings, the methanol extract possesses a significantly higher antioxidant capacity than standard ascorbic acid. Furthermore, the isolated compounds showed strong antioxidant capacity compared to the crude extract and the standard, as illustrated in Figure 9 (Kishore et al., 2018). Among the isolated compounds, mearnsitrin is more effective in antioxidant activity than others with a lower IC_50_ value (Kishore et al., 2018). These findings highlight the effectiveness of isolated compounds over the crude extract, suggesting that bioactive components are primarily responsible for the observed effects. Moreover, the greater potency of these isolated compounds contributes significantly to the diverse biological activities of *M. africana*, reinforcing its therapeutic potential.

### 5.2 Anti-inflammatory activity

Phytochemicals reduce inflammation through multiple mechanisms, including enhancing the release of cytokines, chemokines, and other mediators to induce cellular infiltration, promoting the resolution of inflammation and reestablishment of tissue coordination. At both the cellular and molecular levels, they inhibit enzymes such as cyclooxygenases (COX), lipoxygenases (LOX), phospholipase A2, protein kinases, and phosphodiesterases, which are involved in the production of pro-inflammatory molecules like prostanoids and leukotrienes. Common phytochemicals with anti-inflammatory properties, such as flavonoids, phenolics, alkaloids, saponins, tannins, and terpenes, act by blocking these enzymes or scavenging free radicals, thus inhibiting pro-inflammatory processes (Gonfa et al., 2023; Rajapaksha et al., 2024).

The anti-inflammatory activity of *M. africana* has been investigated in several studies, with varying degrees of efficacy reported for different extracts. Abbhi et al. (2016) investigated *in vivo* the anti-inflammatory activity of methanolic and hydroalcoholic fruit extracts of *M. africana* on carrageenan-induced paw edema. The results showed that both extracts exhibited significant anti-inflammatory activity, with the methanolic extract inhibiting 53% and the hydroalcoholic extract inhibiting 55% of the edema at a dose of 500 mg/kg, after 6 h. This level of inhibition was comparable to ibuprofen, which showed a 74% inhibition in the same model, suggesting that *M. africana* possesses moderate anti-inflammatory properties. In another study, at 500 μg/mL, the aerial parts of *M. africana* were examined for the anti-inflammatory effects of different fractions, including n-hexane and ethyl acetate *in vitro* assay. These fractions showed moderate anti-inflammatory efficacy against human neutrophils, with inhibition rates of 40.9% in n-hexane and 44.8% in ethyl acetate, according to the data in Table 4. In contrast, other fractions and the crude methanolic extract showed lower anti-inflammatory activity at the same concentration, indicating that specific fractions may contribute more significantly to the plant’s anti-inflammatory effects (Ahmad et al., 2011a). The presence of kaempferol, quercetin, and their derivatives in *M. africana* contributes to its anti-inflammatory potential (Gonfa et al., 2023). Overall, *M. africana* exhibits promising anti-inflammatory activity. The potency varies depending on the extract and fraction used. Further research could help identify the active compounds responsible for this activity and explore their potential medicinal applications.

**TABLE 4:** Anti-inflammatory activity of the crude methanol extract and various fractions of the aerial parts of *Myrsine africana* Abbhi et al. (2016).

Test sample/extracts	% Inhibition at 500 μg/mL
Crude methanol	38.5
n-hexane	40.9
Chloroform	37.6
Ethyl acetate	44.8
Butanol	33.1
Aqueous	29.7
Indomethacin	98.0

### 5.3 Antibacterial activity

Phytochemicals exhibit antibacterial activity through various mechanisms, including disruption of bacterial cell membranes, inhibition of protein and enzyme synthesis, and interference with DNA/RNA replication. Terpenoids, alkaloids, phenols and flavonoids disrupt cell membranes, inhibit ATP and protein synthesis, block efflux pumps, and reduce bacterial virulence, positioning them as promising candidates for treating bacterial infections. These compounds damage cell wall integrity, enhance membrane permeability, reduce protein synthesis, and interfere with metabolic enzymes. Flavonoids additionally reduce virulence factor expression, disrupt bacterial energy production, and block efflux pumps, reducing antibiotic resistance, while also causing cell leakage and death, further enhancing their antimicrobial potential (Arrigoni et al., 2024; Huang et al., 2022; Zouine et al., 2024).

In a study conducted by Gul et al. (2021), the antibacterial activity of *M. africana* fruit extracts was evaluated and compared to that of standard antibiotics against various bacterial strains, such as *Staphylococcus aureus*, *E. coli*, *K. pneumoniae*, and *B. subtilis* using agar well diffusion method (Table 5). The fruit extracts demonstrated significant antibacterial effects on both Gram-positive and Gram-negative bacterial strains. These findings suggest that the fruit of *M. africana* contains natural antimicrobial compounds, such as flavonoids, which could be potential agents for treating infectious diseases. The lack of concentrations for determining antibacterial activity is a limitation in these studies. The minimum inhibitory concentration (MIC) values varied between extracts, with methanolic fruit extracts showing the lowest MICs, which may be attributed to the higher purity of bioactive compounds in the methanol solvent (Table 6).

**TABLE 5:** Antibacterial activity of various fruit extracts of *Myrsine africana* Gul et al. (2021).

Bacterial strains	Zone of inhibition (mm) of various fruit extracts				
	Methanol	Ethanol	Chloroform	n-hexane	Aqueous
*S. aureus*	23.5 ± 0.7	19.6 ± 0.6	19.8 ± 0.5	18.3 ± 0.6	11.5 ± 0.6
*E. coli*	22.5 ± 0.97	18.4 ± 0.9	18.7 ± 0.6	18.7 ± 0.5	13.5 ± 0.68
*K. pneumonia*	22.9 ± 0.5	19.5 ± 0.3	15.4 ± 0.6	18.8 ± 0.4	12.7 ± 0.8
*B. subtilis*	21.7 ± 0.3	22.6 ± 0.4	16.6 ± 0.7	17.5 ± 0.8	12.5 ± 0.6
Roxithromycin	13.6 ± 0.9	16.5 ± 0.9	14.5 ± 0.5	13.8 ± 0.5	11.6 ± 0.5
Cefixime	11.5 ± 0.3	12.5 ± 0.3	11.8 ± 0.7	10.8 ± 0.5	10.6 ± 0.7

**TABLE 6:** MICs of *Myrsine africana* fruit extracts (μg/mL) against bacterial strains Gul et al. (2021).

Bacterial strains	MICs of fruit extracts (μg/mL)				
	Methanol	Ethanol	Chloroform	n-hexane	Aqueous
*S. aureus*	0.3 ± 0.1	0.7 ± 0.3	1.8 ± 0.6	1.5 ± 0.3	1.2 ± 0.6
*E. coli*	2.3 ± 0.4	1.5 ± 0.4	1.7 ± 0.5	1.6 ± 0.8	1.6 ± 0.7
*K. pneumonia*	0.7 ± 0.3	0.8 ± 0.3	1.4 ± 0.5	0.9 ± 0.5	1.6 ± 0.4
*B. subtilis*	1.3 ± 0.4	1.6 ± 0.5	1.5 ± 0.7	1.5 ± 0.7	1.8 ± 0.5
Roxithromycin	1.4 ± 0.5	1.3 ± 0.7	1.5 ± 0.3	1.5 ± 0.3	1.5 ± 0.4
Cefixime	1.1 ± 0.7	1.5 ±± 0.5	1.3 ± 0.8	1.3 ± 0.8	1.5 ± 0.5

Similarly, Shopoko et al. (2020) investigated the antibacterial activity of n-hexane, methanol, and aqueous extracts of *M. africana* against *S. aureus* and *E. coli* bacterial strains. The study found that the highest antibacterial activity was showen in the aqueous extracts at 10.0 mg/mL and the methanol extracts at concentrations of 25.0 and 50.0 mg/mL. In comparison, the antibacterial activity of the n-hexane extracts at 25.0 mg/mL and 50.0 mg/mL was significantly lower than that of the aqueous and methanol extracts, as shown in Table 7. The highest activity of the aqueous extract may be attributed to the presence of water-soluble bioactive compounds that are effective against both bacterial strains. In contrast, the antibacterial activity of the methanol extract is likely due to the higher purity and concentration of bioactive compounds such as flavonoids and phenolic acids, which are more effectively extracted in methanol. The lower activity of the n-hexane extract is likely due to the selective nature of n-hexane, which primarily extracts non-polar compounds that may not exhibit higher antibacterial activity.

**TABLE 7:** Antibacterial activity of *Myrsine africana* seed extracts Shopoko et al. (2020).

Extracts	Bacterial strains	% inhibition zone (mm) at different concentration of extracts		
		10.0 mg/mL	25.0 mg/mL	50.0 mg/mL
n-hexane	*S. aureus*	6.0	8.5	12.5
	*E. coli*	6.0	9.0	13.0
Methanol	*S. aureus*	6.5	11.5	18.5
	*E. coli*	6.0	12.5	19.0
Aqueous	*S. aureus*	9.0	11.0	17.5
	*E. coli*	7.0	10.5	16.0

Table 8 demonstrates that the crude methanol extract exhibited the highest antibacterial activity against a broad range of pathogens, significantly outperforming its fractions in most cases at a concentration of 3 mg/mL (Ahmad et al., 2011c). Against *E. coli* (60.86%), *S. typhi* (53.57%), *S. pneumoniae* (57.69%), *P. aeruginosa* (60.00%), *K. pneumoniae* (68.42%), and *B. pumilus* (54.83%), the crude extract consistently showed superior inhibition zones. Notably, the chloroform fraction excelled against *S. aureus* (60.00%) and matched the crude extract’s activity against *K. pneumonia* (68.42%). In contrast, other fractions, including aqueous, butanol, and ethyl acetate, demonstrated limited or no activity against certain pathogens, such as *E. coli*, *B. pumilus*, and *E. aerogenes* (Ahmad et al., 2011c). The study highlighted that the crude methanol extract consistently outperformed other fractions in antibacterial activity against a wide range of bacterial pathogens due to its broad-spectrum activity. However, its superiority over the fractions indicates further research on identifying active compounds, understanding synergy, testing against resistant strains, and optimizing extraction methods to validate the extract’s clinical relevance. The chloroform fraction’s high activity against *S. aureus* is remarkable, but its lack of activity against *E. coli indicates* a need for selective application. The low or no activity observed in other fractions does not diminish their potential; instead, it makes it necessary to optimize the process of finding and isolating bioactive chemicals. The lack of comparison with standard antibiotics limits the interpretation of these findings.

**TABLE 8:** Antibacterial activity of the aerial parts of *Myrsine africana* Ahmad et al. (2011c).

Bacterial strains	% inhibition zone (mm) crude extract and various fractions at 3 mg/mL					
	Crude methanol	n-Hexane	Chloroform	Ethyl acetate	n-Buthanol	Aqueous
*E. coli*	60.86	52.17	—	52.17	47.82	—
*S. epidermidis*	39.39	36.36	33.33	30.30	33.33	36.36
*S. typhi*	53.57	42.82	39.28	46.42	50.0	52.57
*S. pneumoniae*	57.69	50.00	53.84	50.00	50.00	50.00
*S. aureus*	48.0	56.0	60.0	52.0	44.0	48.0
*P. aeruginosa*	60.0	52.0	56.0	48.0	56.0	48.0
*K. pneumoniae*	68.42	63.15	68.42	57.89	47.36	48.38
*B. pumilus*	54.83	45.16	45.16	—	48.33	51.61
*E. aerogenes*	40.62	37.50	37.50	34.37	—	—

Laraib et al. (2021) also investigated the antibacterial properties of *M. africana* fruit and leaf extracts in methanol and chloroform against *S. epidermidis, E. coli,* and *K. pneumonia* bacterial strains. At higher doses, all the extracts significantly increased the antibacterial activity against *S. epidermidis*. The chloroform extract from the fruit displayed the highest activity (83.05%) at 25 mg/mL, while the chloroform and methanol leaf extracts showed 30.94% and 21.39% inhibition, respectively, at the same dosage. Even at 5 mg/mL, the fruit extracts exhibited high percentages of inhibition by methanol and chloroform, at 65.47% and 52.38%, respectively. On the other hand, for Gram-positive bacteria such as *E. coli*, both fruit extracts exhibited a maximum activity of 59.67% at 25 mg/mL, whereas the polar and nonpolar leaf extracts showed maximum inhibition of 28.51% and 21.48%, respectively, at 25 mg/mL. Similarly, the effectiveness of plant extracts against *K. pneumoniae*, another Gram-negative bacterium, did not differ much from that against *E. coli* (Laraib et al., 2021).

These findings collectively suggest that *M. africana* extracts, particularly the methanol and chloroform fractions, possess notable antibacterial properties and could be explored further for their therapeutic potential in treating bacterial infections.

The ethanol extract of *M. africana* inhibited the growth of *E. coli*, *B. cereus*, *S. epidermidis* and *S. pneumoniae* with average zones of inhibition of 22.00 ± 0.00, 20.33 ± 0.33, 25.00 ± 0.00 and 18.17 ± 0.00 mm, respectively. The antibiotic control penicillin showed large zones of inhibition with average of 28.00 ± 0.00, 27.33 ± 0.33, 30.50 ± 0.28 and 25.04 ± 0.87 against *E. coli*, *B. cereus*, *S. epidermidis* and *S. pneumonia*, respectively. The MIC was determined using the agar well diffusion method. The ethanol extracts of *M. africana* showed the best activity with lowest MIC against *E. coli* and *B. cereus*, both with MIC of 7.81 mg/mL. The extract also showed good activity against *S. epidermidis* and *S. pneumoniae*, both with MICs of 15.62 mg/mL. These results have shown that the ethanol extract of *M. africana* has active ingredients against both Gram positive and Gram negative bacteria. The extract also showed MIC values against the organisms that were very low (Obey et al., 2016). The observed results also highlight that ethanol is a more promising solvent for future development due to its safer alternative, providing a balanced combination of efficacy and safety.

### 5.4 Antifungal activity

Phytochemicals exhibit antifungal activity by disrupting fungal cell membranes, inhibiting ergosterol synthesis, and interfering with cell wall biosynthesis. Terpenoids disrupt fungal cell membrane integrity due to their lipophilic nature, causing membrane damage and leakage (Huang et al., 2022). Alkaloids inhibit ergosterol biosynthesis, which is crucial for fungal cell membrane integrity (Wong-Deyrup et al., 2021), while flavonoids hinder fungal growth by disrupting the plasma membrane, inducing mitochondrial dysfunction, and inhibiting cell wall formation, cell division, RNA and protein synthesis, and the efflux pumping system (Aboody and Mickymaray, 2020).

The antifungal activity is likely attributed to bioactive compounds such as flavonoids, tannins, and saponins present in *M. africana*. These compounds may disrupt fungal cell membranes, inhibit spore germination, or interfere with fungal enzyme systems (Aboody and Mickymaray, 2020).

The antifungal activity of *M. africana* exhibits varied efficacy depending on the type of extract, concentration, and fungal strain. A study by Laraib et al., in 2021 examined the antifungal activity of methanol and chloroform extracts from the plant’s fruits and leaves. The methanol fruit extract demonstrated significant inhibition against *Alternaria alternata* (70%–83.64%) and *Aspergillus flavus* (65.39%–78.09%) across concentrations of 5–25 mg/mL. Similarly, the methanol leaf extract showed strong activity, with 83.64% inhibition against *A. alternata* and 72.06% against *A. flavus* at 25 mg/mL. In contrast, the chloroform leaf extract exhibited lower activity, achieving 80% inhibition against *A. alternata* and only 52.06% against *A. flavus* at the same concentration, highlighting the superior efficacy of methanol extracts against fungal strains. Conversely, another study using the agar tube dilution method found that crude methanolic extracts and fractions exhibited low activity, with the highest inhibition being 24% against *Fusarium oxysporum* (aqueous fraction) and minimal to no activity against fungi such as *Aspergillus niger* and *Rhizopus stolonifer* (Ahmad et al., 2011b). These findings collectively highlight that the antifungal efficacy of *M. africana* may depend on extraction methods and test conditions, warranting further research is needed to optimize extraction methods, explore the mechanisms behind the observed antifungal effects, and isolate the active compounds responsible for these activities.

### 5.5 5-Lipoxygenase (5-LOX) inhibition

5-Lipoxygenase (5-LOX) is a crucial enzyme in the biosynthesis of pro-inflammatory leukotrienes, especially LTB4, which plays a crucial role in inflammation, immune responses, and bronchoconstriction. Overproduction of leukotrienes is associated with chronic inflammatory diseases such as asthma, rheumatoid arthritis, and cardiovascular diseases, making 5-LOX a promising target for therapeutic intervention (Kahnt et al., 2022; Tian et al., 2020; Wang et al., 2021). Inhibition of 5-LOX or blocking leukotriene synthesis has shown potential in managing these conditions (Tian et al., 2020).

Phytochemicals including flavonoids, tannins, saponins, and alkaloids, inhibit 5-LOX activity. These compounds modulate 5-LOX through various mechanisms, such as directly binding to the enzyme, reducing oxidative stress, altering cellular membrane dynamics, and modulating inflammatory signaling pathways like nuclear factor kappa B (NF-kB), which downregulates 5-LOX expression (Gonfa et al., 2023; Mukhopadhyay et al., 2023). The combined actions of these phytochemicals suggest that plant extracts, such as *M. africana*, hold promise as therapeutic agents for treating inflammatory disorders, including arthritis, asthma, and cardiovascular diseases. The crude ethanol extract of *M. africana* and its isolated compound myrsinoside B were evaluated for their ability to inhibit 5-LOX. Both the crude extract and myrsinoside B demonstrated 5-LOX inhibition with IC_50_ values of 29.65 ± 2.92 μg/mL and 29.23 ± 3.08 μg/mL, respectively (Fibrich et al., 2019). The IC_50_ values for both the crude extract and myrsinoside B were comparable, suggesting that these compounds could be used to manage inflammatory diseases. However, the inhibition was less potent than that of the standard caffeic acid (IC_50_ value of 14.87 ± 0.69 μg/mL), indicating that while promising, and further research is needed to optimize these compounds’ anti-inflammatory potential. Ongoing studies into plant-derived 5-LOX inhibitors, including those from *M. africana*, are expected to enhance their clinical application for managing chronic inflammatory diseases and potentially cancer, where leukotriene signaling contributes to tumor growth and metastasis.

### 5.6 Cytotoxicity

Phytochemicals exert cytotoxicity through multiple mechanisms, including apoptosis induction, cell cycle arrest, DNA damage, autophagy modulation, inhibition of angiogenesis, and the disruption of metastasis. These mechanisms highlight the therapeutic potential of phytochemicals, particularly in cancer treatment, by targeting cancer cell survival and proliferation pathways (Choudhari et al., 2020; Majrashi et al., 2023).

*In vitro* cytotoxicity studies on human keratinocytes were conducted using concentrations ranging from 400 to 3.5 μg/mL over 72 h. The finding revealed that the ethanol extract of *M. africana* and the isolated compound myrsinoside B were nontoxic at the highest dose tested (400 μg/mL). The IC_50_ value for Actinomycin D, the positive control, was less than 5 × 10^−3^ μg/mL, significantly lower than the lowest concentration tested for the extracts, confirming their safety profile in this context (Fibrich et al., 2019). In a related study, Stapelberg et al. (2019) examined the cytotoxic activity of ethanol leaf extract on mouse melanocyte B16-F10 cells, yielding an IC_50_ value of 155.4 μg/mL. In comparison, the standard Actinomycin D showed an IC_50_ value of 9.15 × 10^−3^ μg/mL, emphasizing the relative lack of potency of the leaf extract in this context. While the leaf extract displayed some cytotoxic potential, its higher IC_50_ value suggests a lower efficacy when compared to the potent positive control.

In contrast, Laraib et al. (2021) conducted cytotoxicity studies on methanol and chloroform extracts from the leaf and fruit of *M. africana* using the Brine Shrimp Lethality Test and the potato disk assay. The results indicated that the methanol and chloroform extracts of the fruit achieved 100% brine shrimp lethality at concentrations of 1,000 and 500 μg/mL, respectively. In contrast, the leaf extracts demonstrated lower lethality, with 33.33% for the methanol extract and 20% for the chloroform extract at a tested concentration of 1,000 μg/mL. The LD_50_ values for the fruit extracts were significant, measuring 49.86 μg/mL for methanol and 226.39 μg/mL for chloroform, indicating considerable cytotoxic potential. In comparison, the LD_50_ values for the leaf extracts were notably higher: 1,377.5 μg/mL for methanol and 2,193.15 μg/mL for chloroform, suggesting that the fruit is more biologically active than the leaf. For reference, the standard compounds etoposide, potassium dichromate, and vincristine had LD50 values of 7.46 μg/mL, 7.46 μg/mL, and 0.397 μg/mL, respectively. The highest tumor inhibition observed was 82.22% and 81.85% for the methanol and chloroform fruit extracts, respectively, at a tested concentration of 1,000 μg/mL. At the lowest tested concentration of 10 μg/mL, tumor inhibition rates were 50.37% and 39.62% for the methanol and chloroform extracts, respectively. The methanol and chloroform leaf extracts exhibited tumor inhibition of 53.70% and 49.62% at 1,000 μg/mL, respectively. Generally, a tumor inhibition rate of 20% is considered significant for plant extracts. The fruit extracts displayed notable cytotoxic activity compared to the leaf extracts, likely due to a higher concentration of saponins and phenolic compounds, believed to contribute to the observed cytotoxic effects. Additionally, the variation in antitumor activity may be from the differing bioactive compounds present in various parts of the plant and the solvents employed for extraction (Laraib et al., 2021).

Kabubii et al. (2015) conducted *in vivo* acute toxicity studies on the aqueous seed extract of *M. africana* in male Wistar rats, using doses of 1,000 and 5,000 mg/kg body weight. The findings revealed that the median lethal dose (LD_50_) exceeds 5,000 mg/kg, as no animals died during the 14-day study period at the tested doses. Additionally, no significant changes in body weight were observed compared to the control group, suggesting a relatively low toxicity profile. Hematological analyses revealed that red blood cell (RBC) count and packed cell volume (PCV) were significantly elevated in the 5,000 mg/kg treatment group at 48 h but normalized by day 14. Hemoglobin concentration (HB), mean cell volume (MCV), mean corpuscular hemoglobin (MCH), and mean corpuscular hemoglobin concentration (MCHC) showed no significant differences between the treatment groups and the control, indicating that the extract did not induce anemia. Biochemical analyses revealed a significant increase in aspartate aminotransferase (AST) levels in both treatment groups by day 14.

In contrast, alanine aminotransferase (ALAT) levels did not significantly differ between treated and control groups, suggesting that the extract may not cause substantial liver toxicity, as ALAT is more specific to liver injury. These studies revealed that the aqueous seed extract of *M. africana* has relatively low toxicity, with no significant changes in hematological or biochemical parameters at the tested doses. These findings support the potential safe for therapeutic applications at certain doses. However, further studies are needed to confirm its long-term safety and to establish effective doses for specific therapeutic purposes. Notably, doses lower than 1,000 mg/kg body weight are recommended for ensuring safety in clinical use (Kabubii et al., 2015).

These studies collectively highlight the promising cytotoxic and antitumor potential of *M. africana*, particularly its fruit extracts. The favorable safety profiles observed *in vitro* and in acute toxicity studies suggest that there are potential therapeutic applications, warranting further exploration and use in traditional medicine.

## 6 Conclusion and future prospects

In conclusion, *M. africana* has emerged as a rich source of phytochemicals with diverse biological activities, showing great potential for the development of novel therapeutic agents. However, the current research is limited, and further comprehensive studies are necessary to fully explore its medicinal potential and facilitate its utilization in the healthcare and pharmaceutical industries. Specific research gaps include investigating the mechanisms of action of isolated compounds, which is crucial for understanding of their therapeutic potential at the molecular level. Additionally, conducting clinical studies are necessary to assess the safety, efficacy, and optimal dosage of *M. africana*-derived products for human use. Bioavailability studies are also needed to evaluate how the bioactive compounds are absorbed, distributed, and metabolized in the body. It is equally important to examine potential synergistic effects between different phytochemicals in plant extracts, as this could enhance their therapeutic efficacy. It is equally important to examine the potential synergistic effects between different phytochemicals in plant extracts, as this could enhance their therapeutic efficacy. Moreover, sustainable harvesting and cultivation practices should be adopted to conserve *M. africana* and ensure a steady supply of medicinal resources, especially in light of its growing demand. Overall, while *M. africana* holds great promise, addressing these research gaps will be vital for advancing its practical applications in medicine.
